# Attitudes Towards Brain Health and Dementia Risk Reduction Among Minoritised Ethnic Communities in the United Kingdom: A Cross‐Sectional Survey Analysis

**DOI:** 10.1002/gps.70238

**Published:** 2026-07-11

**Authors:** Golnaz L. Atefi, Charles R. Marshall, Claudia Cooper, Sube Banerjee, Naaheed Mukadam, DeNPRU‐QM

**Affiliations:** ^1^ Department of General Practice Amsterdam UMC Amsterdam the Netherlands; ^2^ Division of Psychiatry University College London London UK; ^3^ Centre for Preventive Neurology Queen Mary University of London London UK; ^4^ Barts Health NHS Trust London UK; ^5^ Centre for Psychiatry and Mental Health Wolfson Institute of Population Health Queen Mary University of London London UK; ^6^ Faculty of Medicine and Health Sciences University of Nottingham Nottingham UK; ^7^ North London NHS Foundation Trust London UK

**Keywords:** attitude, brain health, dementia literacy, dementia prevention, ethnic minority communities, risk factor awareness, stigma

## Abstract

**Background:**

There is much excitement about the potential for dementia prevention by targeting modifiable risk factors, yet sociodemographic disparities in dementia‐related knowledge, stigma, and risk factor awareness remain underexplored. This study examines these differences among minoritised ethnic communities in the UK.

**Methods:**

As part of a project by the NIHR Dementia and Neurodegeneration Policy Research Unit at Queen Mary (DeNPRU‐QM), dementia knowledge, stigma, and risk factor awareness of 3500 participants were assessed in a secondary analysis of data from two national surveys conducted in 2023 by Alzheimer's Research UK. Linear regression models were used to examine associations between ethnicity and knowledge‐related outcomes, while ordinal logistic regression was used for stigma, adjusting for age, sex, education, social grade, chronic conditions, and knowing someone with dementia.

**Results:**

Compared to White participants, South Asian (Coef. = −0.45, 95% CI [−0.55, −0.34], *p* < 0.001) and Black participants (Coef. = −0.17, 95% CI [−0.27, −0.06], *p* < 0.005) had lower knowledge about dementia. Compared to White participants, Black participants had higher odds of reporting greater stigma (OR = 2.55, 95% CI [2.10, 3.09], *p* < 0.001), as did South Asian participants (OR = 1.62, 95% CI [1.34, 1.97], *p* < 0.001) and participants from Other ethnic groups (OR = 1.55, 95% CI [1.17, 2.07], *p* = 0.003). Black individuals also had lower knowledge of dementia risk factors compared to White participants (Coef. = −0.655, 95% CI [−1.110, −0.200], *p* = 0.005). Education level, chronic health conditions, gender, profession, and knowing someone with dementia were also associated with some outcomes, although associations varied by outcome.

**Conclusion:**

Ethnic disparities exist in dementia knowledge, stigma, and dementia risk factor awareness. Any future public information campaign around dementia risk reduction and timely diagnosis should ensure cultural competency and include strategies to reach minoritised ethnic communities. Policymakers should consider how prevention might be promoted in all health and social care encounters, since people with chronic conditions who may not be eligible for primary health checks had lower levels of knowledge about dementia.

## Key Points

- Ethnic disparities in dementia knowledge are evident even before dementia diagnosis or direct care involvement.
- Disclosure‐related stigma is higher among minoritised ethnic groups.
- Dementia risk‐factor awareness and broader brain‐health knowledge are not the same construct.
- Equitable dementia prevention requires more than generic public information campaigns.

## 1 Introduction

Primary prevention has the aim of reducing the incidence of disease and will be central to health policy in the next decade [1, 2]. In England, UK, the NHS Health Check programme launched in 2009, which screens healthy individuals aged 40–74 for risk for heart disease, stroke, diabetes, and kidney disease, has been linked to reduced disease incidence across multiple organ systems [3].

Dementia is a major global health issue affecting approximately 50 million people worldwide, with this number estimated to triple by 2050 [4]. There is substantial evidence supporting the potential for risk factor modification to prevent or delay the onset of dementia [5, 6]. These risk factors include healthy lifestyle behaviours, such as not smoking, regular physical activity, reduced alcohol consumption and social contact [7].

There have been national initiatives which have sought to inform the UK public about dementia risk factors. In 2018, information about seeking help for memory symptoms was added to the English NHS Health Check programme for those aged over 65, although this programme's primary focus remains cardiovascular disease prevention. In 2023, a UK charity, Alzheimer's Research UK (ARUK) launched the Think Brain Health online tool to enable people to explore how to reduce their dementia risk; to date, half a million people have completed it [3].

Attitude is one of the key factors associated with lifestyle choices associated with dementia risk, along with environmental factors that also play a pivotal role [8, 9]. It is defined as a tendency to respond favourably or unfavourably to specific concepts or behaviours, including lifestyle choices and health promotion behaviours, and can be an indicator of stigma [8, 9]. These attitudes are likely to vary for sociocultural reasons in different ethnic groups. Successful dementia prevention strategies may benefit from accounting for prevailing attitudes, including beliefs around the preventability of dementia [10, 11], but most research exploring such attitudes has been conducted in individuals of European origin [1]. This is an important evidence gap, because minoritised ethnic groups are at higher risk of dementia than White individuals, due to factors including a higher burden of modifiable risk factors such as diabetes [12, 13].

Recent evidence indicates variation in dementia‐related knowledge and attitudes across ethnic groups within the UK and similar contexts. For example, Cheston et al. 2025 study on dementia attitudes in UK Chinese population highlights differences in willingness to disclose dementia‐related concerns and engagement with brain health information [14]. Population‐based and survey research also suggests that awareness of dementia risk factors and beliefs about preventability vary across sociodemographic groups, including ethnicity, education, and exposure to dementia [15, 16, 17]. However, most existing studies examine single populations or individual aspects of dementia knowledge.

Nearly one fifth of the UK population comes from a minority ethnic background, with South Asian (including Bangladeshi, Indian, Pakistani, and Sri Lankan individuals) and Black communities (African or Afro‐Caribbean) the largest groups [13]. Dementia prevention approaches need to be culturally competent, to ensure that they reach under‐served, minoritised communities. If disparities in attitudes towards dementia exist, attainment of health equity may require additional, tailored approaches, which consider different attitudes and informational needs, as well as other factors that may operate differently in under‐served communities, including digital exclusion and language barriers [18].

The NIHR Dementia and Neurodegeneration Policy Research Unit at Queen Mary (DeNPRU‐QM) was commissioned by the Department of Health and Social Care (DHSC) in 2023 to explore public attitudes to dementia prevention. We have previously reported that almost half of the English public reported having some knowledge of dementia prevention and considered how current evidence should inform policy approaches [19]. In this study, we aim to compare the beliefs and attitudes towards brain health and dementia in South Asian and Black communities, with those of the White population, taking into account socioeconomic variation, to inform future policy approaches to enable equitable prevention. In this study, three related but conceptually distinct constructs are examined [1]: dementia knowledge, referring to general beliefs about dementia and its relationship to ageing and modifiability [2]; dementia risk factor awareness, referring to knowledge of specific modifiable risk factors associated with dementia; and [3] brain health knowledge, referring to broader perceptions of factors associated with overall brain health, which may not always be explicitly linked to dementia. These constructs reflect different levels of abstraction and are considered separately.

## 2 Methods

### 2.1 Recruitment

We analysed data aggregated from two surveys carried out on behalf of Alzheimer's Research UK (ARUK), a UK‐based dementia research charity and policy influencing organisation. The first dataset was taken from a biannual survey, which aimed to assess attitudes to dementia, carried out by Ipsos, a market research company. Ipsos surveyed a nationally representative quota sample of 2530 adults in the United Kingdom aged 18+, using its telephone omnibus survey between 7 June and 4 July 2023. There was an ethnic minority boost within this, resulting in a total sample of 604 adults from minority ethnic backgrounds. The survey data was weighted to the known national population proportions of this group for age within gender, government office region within gender, working status, ethnicity, and social grade. Data from this survey are published online [20].

The second survey considered attitudes towards brain health and was conducted online by insight agency Opinium, commissioned by Claremont Communications on behalf of Alzheimer's Research UK. It was available online from 9th to 22nd January 2024. The focus was on identifying behavioural insights to inform future brain health campaigns targeting under‐served audiences. There were ethnic boosts for respondents of Chinese and mixed ethnicity. Results were weighted to be nationally representative.

This aggregate data was provided by Alzheimer's Research UK in anonymised form, so further ethical approval was not required for this study. All participants provided informed consent to participate in the study, either online or by telephone. Patient and public involvement was integrated into this study to support the interpretation of findings. The results were discussed with contributors to ensure that the conclusions reflected lived experience and were relevant to real‐world contexts. Input from two public contributors, one with lived experience of dementia and one from a minoritised ethnic background, was used to contextualise the findings.

### 2.2 Data Collection

The first survey was developed by ARUK in partnership with lay and academic advisors and is run every 2 years to measure attitudes to dementia, including perceptions of dementia, stigma, and awareness of dementia risk. Example items include statements such as ‘Brain problems, such as dementia, are a natural consequence of old age’. The second survey was commissioned by ARUK to inform their Think Brain Health campaign and focussed on broader perceptions of brain health and lifestyle‐related factors. Example items include questions such as ‘How much of an impact, if any, do you think the following have on brain health?’ (e.g., diet, physical activity, sleep). While both surveys address related concepts, the first primarily focuses on dementia‐specific knowledge and stigma, whereas the second captures broader awareness of brain health and perceived impact of modifiable lifestyle factors. Survey questions are provided in Appendix 1 (Table A1).

### 2.3 Ethnicity Classification

Ethnicity was self‐reported using categories aligned with the England Census (Office for National Statistics (ONS) [21, 22]. Participants self‐identified their ethnic group and we grouped them into one of the following supraordinate census categories: White (including English, Welsh, Scottish, Northern Irish, British, Irish, Gypsy or Irish Traveller, and other White backgrounds); Black (including African, Caribbean, and other Black, African, or Caribbean backgrounds); South Asian (including Indian, Pakistani, and Bangladeshi); or Other (encompassing mixed ethnicities and any other specified ethnic background).

### 2.4 Confounders

Sociodemographic data were collected through self‐reported survey questions, including age, sex, education level, social grade, chronic condition, and knowing someone with dementia. Age was modelled as a continuous variable in years, and sex was coded as male/female, with male as the reference category. Education was assessed as the highest academic or professional qualification completed and coded ordinally as non‐graduate, other qualification, and graduate. Social grade was classified based on the UK Office for National Statistics occupational classifications and categorised into [1]: higher and intermediate managerial, administrative, and professional occupations; and [2] skilled, semi‐skilled, and unskilled manual workers, as well as unemployed individuals [23]. The skilled, semi‐skilled and unskilled manual/unemployed group was used as the reference category. Additionally, participants were asked whether they had a long‐term chronic condition and whether they knew someone with dementia; these variables were coded as no versus yes, with no chronic condition and not knowing someone with dementia used as the respective reference categories.

### 2.5 Main Outcomes

Further detail on outcome measures (survey questions) can be found in Appendix 1. The study assessed general dementia knowledge, stigma, dementia risk factor awareness, and brain health knowledge. These were derived from different survey instruments and analysed separately.

### 2.6 Knowledge About Dementia (Data From Surveys 1 and 2 Combined)

This was assessed through two key survey questions, evaluating participants' understanding of whether dementia is a natural consequence of ageing and whether brain health can be protected. Responses were rated on a scale from 0 to 2, with 0 indicating lower knowledge, 1 for uncertain knowledge, and 2 for higher knowledge and awareness of dementia. This variable is interpreted as a proxy measure of general dementia‐related knowledge.

### 2.7 Stigma (Data From Surveys 1 and 2 Combined)

Stigma related to dementia was assessed by evaluating participants' comfort in discussing concerns about possible symptoms of dementia with others. Responses were rated on a scale from 0 to 2, with 0 indicating comfort, 1 for uncertainty, and 2 for discomfort. Higher scores reflected greater stigma around discussing dementia‐related concerns, while lower scores indicated less stigma or reluctance to disclose potential symptoms. This single‐item measure captures willingness to disclose a diagnosis and is interpreted as a proxy indicator of stigma.

### 2.8 Knowledge About Dementia Risk Factors (Survey 1)

This measure focuses on the identification of predefined specific risk factors. This variable operationalises awareness of specific dementia risk factors and does not encompass broader constructs of brain health knowledge. Knowledge about dementia risk was assessed based on asking participants whether they thought dementia risk could be reduced and then testing their awareness of modifiable risk factors [3]. Participants were asked whether they believed dementia risk could be reduced using specific strategies, for example by modifying diet, physical activity, sleep, mental well‐being, cognitively stimulating activities, social contact, alcohol consumption, smoking, hearing loss, cholesterol levels, blood pressure, and blood sugar levels. Responses were scored as 2 for “Yes” and 0 for “No.” Responses for all 13 questions were summed to give a possible score of between zero and 26.

### 2.9 Knowledge About Brain Health (Survey 2)

Participants were asked if people could reduce their dementia risk. They also rated the impact of various modifiable factors on brain health: diet, physical activity, sleep, mental well‐being, cognitively stimulating activities, social contact, alcohol consumption, smoking, hearing loss, cholesterol levels, blood pressure, and blood sugar levels. Responses were categorized as two if the factor was perceived to have a major or moderate impact on brain health and 0 if it was believed to have a small or no impact. The total knowledge score was calculated by summing individual item scores (ranging from 0 to 26), providing an overall measure of participants' understanding of dementia risk and the role of lifestyle and health factors in brain health. This measure reflects the perceived importance of factors associating with brain health and does not necessarily indicate explicit knowledge of dementia prevention. Therefore, this measure is interpreted as a proxy indicator of general brain health awareness.

### 2.10 Statistical Analysis

Socio‐demographic variables and questionnaire outcomes were analysed using descriptive and frequentist statistical methods in STATA [24]. Continuous data were summarized by mean and standard deviation, and categorical data were presented as counts and percentages across the different ethnic groups. Responses such as “I don't know,” “prefer not to answer,” or blank responses were treated as missing. Analyses were conducted using complete case analysis, excluding observations with missing data on exposure, outcomes, or covariates. Given the low proportion of missing data (< 5% across variables), this approach was considered unlikely to substantially bias estimates; however, the potential for bias due to non‐random missingness cannot be excluded.

Survey data were provided in weighted form by the data collection agencies to reflect national population distributions. Analyses were conducted using these pre‐weighted datasets, and no additional weighting procedures were applied. We used appropriate descriptive statistics to summarize knowledge about dementia, stigma, knowledge about brain health factors, and knowledge about dementia risk factors across ethnic groups. To ensure consistency in the analysis, the datasets from both surveys were merged for equivalent survey questions. For variables common to both surveys, datasets were combined by harmonising variable coding and response categories prior to analysis. Only equivalent items with identical or directly comparable wording were merged. Variables that differed conceptually or in measurement (e.g., dementia risk factor awareness and brain health knowledge) were analysed separately within their respective survey datasets.

We used linear regression models to explore factors associated with the knowledge‐related outcome measures, including knowledge about dementia, knowledge about dementia risk factors, and knowledge about brain health, with ethnicity as the main exposure. Linear regression assumptions were assessed through standard diagnostic plots. Multicollinearity between covariates was evaluated with no evidence of problematic collinearity observed. Because stigma was assessed using a single ordinal item coded from 0 = low stigma to 2 = high stigma, ordinal logistic regression was used for this outcome. For the ordinal logistic regression model, results are presented as odds ratios, with odds ratios above one indicating higher odds of reporting greater stigma. All models were adjusted for age, sex, social grade, education, having a chronic health condition, and knowing someone with dementia. To account for multiple statistical tests, we applied a Bonferroni correction by dividing the usual significance level by the number of regression models run [25]. Consequently, the *p* value threshold below which the null hypothesis would be rejected was 0.0167.

## 3 Results

### 3.1 Sample Description

The sociodemographic characteristics of the combined dataset are presented below in Table 1 and a detailed breakdown of the sociodemographic information for each survey is presented in Appendix 2 (Table A2 and Table A3).

**TABLE 1:** Description of the socio‐demographic characteristics of the study sample by ethnicity.

	White (*N* = 1897)	South Asian (*N* = 680)	Black (*N* = 664)	Other (*N* = 259)	Overall (*N* = 3500)
Age					
Mean (SD)	51.78 (17.05)	45.15 (10.38)	44.91 (10.81)	42.48 (14.30)	48.50 (15.12)
Range min‐max	18–97	18–78	18–83	18–92	18–97
Missing	49 (2.65%)	14 (2.10%)	21 (3.26%)	6 (2.37%)	90 (2.57%)
Gender					
Male	952 (50.18%)	328 (48.24%)	309 (46.47%)	133 (51.35%)	1722 (49.19%)
Female	922 (48.60%)	346 (50.88%)	351 (52.78%)	121 (46.72%)	1740 (49.70%)
Missing	23 (1.21%)	4 (0.59%)	4 (0.60%)	5 (1.93%)	36 (1.03%)
Skilled, semi‐skilled and unskilled professions	576 (29.89%)	200 (29.41%)	208 (31.28%)	50 (19.31%)	1025 (30.2%)
Higher/intermediate managerial, administrative and professional occupations	1262 (66.53%)	468 (68.82%)	443 (66.62%)	192 (74.13%)	2365 (69.7%)
Missing	68 (3.58%)	12 (1.76%)	14 (2.11%)	17 (6.56%)	111 (3.17%)
Non graduate	857 (45.18%)	239 (35.15%)	233 (35.04%)	84 (32.43%)	1413 (40.36%)
Other education	152 (8.01%)	18 (2.65%)	17 (2.56%)	11 (4.25%)	198 (5.66%)
Graduate	854 (45.02%)	422 (62.06%)	414 (62.26%)	160 (61.78%)	1850 (52.84%)
Missing	34 (1.79%)	1 (0.15%)	1 (0.15%)	4 (1.54%)	40 (1.14%)
Not having a chronic condition	1121 (59.09%)	429 (63.09%)	433 (65.11%)	172 (66.41%)	2155 (61.55%)
Having a chronic condition	759 (40.01%)	243 (35.74%)	225 (33.83%)	79 (30.50%)	1306 (37.30%)
Missing	17 (0.90%)	8 (1.18%)	7 (1.05%)	8 (3.09%)	40 (1.14%)
Not knowing someone with dementia	815 (42.96%)	386 (56.76%)	414 (62.26%)	141 (54.44%)	1756 (50.16%)
Knowing someone with dementia	1075 (56.67%)	262 (38.53%)	223 (33.53%)	114 (44.02%)	1674 (47.81%)
Missing	7 (0.37%)	32 (4.71%)	28 (4.21%)	4 (1.54%)	71 (2.03%)

Ethnicity data was missing for 30 participants, so the final sample consisted of a total of 3500 participants, of whom 3027 lived in England. They self‐identified as coming from: White (*N* = 1897), South Asian (*N* = 680), Black (*N* = 664), and Other (*N* = 259) ethnic groups. The overall mean age was 48.5 years (SD = 15.1), with White participants being older than the other ethnic groups. Gender distribution was relatively balanced across groups (50.2% male participants). Overall, 69.7% of participants were from higher and 30.2% from lower professional groups; this was consistent across ethnic groups. A substantial portion of participants (52.8% overall) across all ethnic groups had completed a university degree. White participants had 45.0% graduates, compared to 62.0% in the South Asian group, 62.2% in the Black group, and 61.7% in the Other and mixed ethnic group. Overall, 37.3% of participants reported having a chronic condition; this was highest in the White group (40.0%); 35.7% in the South Asian and 33.8% in the Black group and 30.5% in the Other group. About 50.1% of participants reported knowing someone with dementia. Minority ethnic groups were less likely to know someone with dementia compared to the White group. Overall, proportion of missing data was low across all groups (< 5% in each category). Table 1 provides an overview of the descriptive statistics for socio‐demographic data by ethnicity.

## 4 Summary Statistics of Outcome Measures

Summary scores for all outcome measures are shown in Table 2. Results are presented separately for each outcome domain. The mean knowledge score about dementia across all ethnic groups was 3.06 (SD = 1.1), with some variations across ethnic groups. The White participants had the highest mean score of knowledge about dementia (3.19, SD = 1.0) compared to others, while the South Asian group in this study reported the lowest level at 2.77 (SD = 1.2) (see Table 2).

**TABLE 2:** Descriptive statistics for outcome measures across ethnic groups.

Combined data	White (*N* = 1897)	South Asian (*N* = 680)	Black (*N* = 664)	Other (*N* = 259)	Overall (*N* = 3500)
Knowledge about dementia/Mean (SD)	3.19 (1.02)	2.77 (1.25)	3.07 (1.15)	2.96 (1.16)	3.06 (1.12)
Missing	66 (3.48%)	7 (1.03%)	11 (1.65%)	15 (5.79%)	99(2.83%)
Stigma/Mean (SD)	0.59 (0.84)	0.84 (0.96)	1.04 (0.98)	0.82 (0.94)	0.74 (0.92)
Missing	50 (2.64%)	19 (2.79%)	16 (2.41%)	4 (1.54%)	89 (2.54%)
Survey 1	White (*N* = 1897)	Asian (*N* = 210)	Black (*N* = 210)	Other (*N* = 183)	Overall (*N* = 2500)
Knowledge about dementia risk factors/Mean (SD)	2.45 (2.92)	2.56 (3.33)	1.78 (2.70)	2.27 (2.70)	2.39 (2.93)
Missing	0	0	0	0	0
Survey 2		South Asian (*N* = 470)	Black (*N* = 454)	Others (*N* = 76)	Overall (*N* = 1000)
Knowledge about brain health/Mean (SD)		20.96 (5.94)	20.92 (5.69)	20.13 (6.23)	20.88 (5.85)
Missing		0	0	0	0

The White group had the lowest mean stigma of 0.59 (SD = 0.8), suggesting that participants felt relatively comfortable discussing concerns about possible symptoms of dementia with others. The Black group had the highest mean stigma score of 1.04 (SD = 0.9), followed by the Other group (0.8, SD = 0.9) and South Asian participants (0.8, SD = 0.9).

Knowledge about dementia risk factors was evaluated in 2500 participants, including White (*N* = 1897), Asian (*N* = 210), Black (*N* = 210), and Other (*N* = 183) ethnic groups. The findings suggest limited knowledge across all ethnic groups, with some variation between them. The South Asian group had the highest mean score of 2.56 (SD = 3.3), while the Black group had the lowest mean score of 1.78 (SD = 2.7). The White and Other groups had mean scores of 2.45 (SD = 2.9) and 2.27 (SD = 2.7), respectively. Overall, the mean score across all groups was 2.39 (SD = 2.9), and there were no missing data.

Knowledge about brain health was evaluated across minority ethnic groups only, with a total of 1000 participants, including South Asian (*N* = 470), Black (*N* = 454), and Other (*N* = 76) ethnic groups. Overall, participants in all ethnic groups demonstrated relatively high and similar level of knowledge about brain health. The South Asian group had a mean score of 20.9 (SD = 5.9), closely followed by the Black group with a mean score of 20.9 (SD = 5.6). The Other group had a mean score of 20.1 (SD = 6.2), and the overall mean score across all groups was 20.8 (SD = 5.8).

### 4.1 Multivariable Analyses

We show adjusted regression analyses in Table 3. Unadjusted regression results, which showed associations between ethnicity and all three outcomes of interest: knowledge about dementia, stigma and knowledge about dementia risks are shown in Appendix 3. Linear regression was used for knowledge‐related outcomes, while ordinal logistic regression was used for stigma.

**TABLE 3:** Regression analysis adjusted for Ethnicity, Age, Gender, Social Grade, Education, Chronic Condition and Knowing Someone with Dementia.

Outcome		Covariate	Estimate	Lower confidence interval	Upper confidence interval	*p*‐value
Knowledge about dementia	Ethnicity	White	Reference			
		South Asian	−0.447	−0.552	−0.343	< 0.001
		Black	−0.167	−0.273	−0.06	0.002
		Others	−0.224	−0.384	−0.065	0.006
	Age		−0.003	−0.005	0.001	0.064
	Female sex		0.061	−0.011	0.133	0.097
	Profession		0.216	0.126	0.306	< 0.001
	Education		0.099	0.056	0.142	< 0.001
	Chronic condition		−0.132	−0.213	−0.051	0.001
	Knowing someone with dementia		0.068	−0.012	0.147	0.094
Stigma	Ethnicity	White	Reference			
		South Asian	1.622	1.338	1.967	< 0.001
		Black	2.545	2.095	3.092	< 0.001
		Others	1.553	1.167	2.067	0.003
	Age		0.999	0.994	1.004	0.586
	Female sex		1.187	1.028	1.370	0.020
	Profession		1.000	0.847	1.181	0.999
	Education		1.103	1.018	1.194	0.016
	Chronic condition		1.250	1.076	1.453	0.004
	Knowing someone with dementia		0.911	0.787	1.055	0.213
Knowledge about dementia risk factors	Ethnicity	White	Reference			
		South Asian	−0.157	−0.602	0.289	0.491
		Black	−0.655	−1.11	−0.2	0.005
		Others	−0.257	−0.737	0.223	0.294
	Age		−0.005	−0.012	0.002	0.174
	Female sex		0.158	−0.052	0.369	0.14
	Profession		0.335	0.063	0.607	0.016
	Education		0.551	0.421	0.681	< 0.001
	Chronic condition		−0.116	−0.367	0.135	0.365
	Knowing someone with dementia		0.39	0.149	0.63	0.002

*Note:* Linear regression was used for knowledge‐related outcomes, and ordinal logistic regression was used for stigma. For knowledge‐related outcomes, estimates are coefficients from linear regression models. For stigma, estimates are odds ratios from ordinal logistic regression. Reference categories were White ethnicity, male sex, lower occupational social grade, no chronic condition, and not knowing someone with dementia. Age was modelled as a continuous variable in years. Education was modelled ordinally as non‐graduate, other qualification, and graduate.

In the adjusted regression (Table 3), all minority ethnic groups reported lower knowledge about dementia and higher stigma, compared with the White majority reference group. Greater dementia knowledge was also associated with coming from a managerial/intermediate professional socioeconomic group and having received more years of education. Having a chronic condition was associated with reporting less dementia knowledge.

In the ordinal logistic regression model for stigma, all minoritised ethnic groups had higher odds of reporting greater stigma compared with White participants. Black participants had the highest odds of reporting greater stigma (OR = 2.55, 95% CI [2.10, 3.09], *p* < 0.001), followed by South Asian participants (OR = 1.62, 95% CI [1.34, 1.97], *p* < 0.001) and participants from Other ethnic groups (OR = 1.55, 95% CI [1.17, 2.07], *p* = 0.003). Having a chronic condition was also associated with higher odds of reporting greater stigma (OR = 1.25, 95% CI [1.08, 1.45], *p* = 0.004). Education was associated with slightly higher odds of reporting greater stigma (OR = 1.10, 95% CI [1.02, 1.19], *p* = 0.016). Female sex was associated with higher odds of reporting greater stigma compared with male sex, although this association did not meet the Bonferroni‐corrected threshold for statistical significance. The adjusted regression results also revealed ethnic group differences in knowledge about dementia risk factors. Specifically, Black individuals had lower knowledge compared to White individuals.

Managerial, administrative and professional occupations, and higher education were both positively associated with greater knowledge. Additionally, knowing someone with dementia was positively associated with increased knowledge, meaning that individuals who had personal connections with someone affected by dementia tended to have greater knowledge about dementia risk factors.

### 4.2 Input From Patient and Public Involvement

The public contributors highlighted that cultural relevance, representation, and the use of inclusive language are central to increasing engagement and trust in dementia prevention efforts. They noted that research and health communication should avoid stigmatising or blame‐oriented language around lifestyle and dementia, and instead reflect the values and self‐perceptions of the communities involved, with particular emphasis on trust‐building in community engagement. The discussion also drew attention to structural barriers such as digital exclusion and underrepresentation in the healthcare workforce, underscoring the need for interventions that are both culturally responsive and accessible.

## 5 Discussion

This study examined general dementia knowledge, stigma, dementia risk factor awareness, and brain health knowledge across different ethnic groups. White participants demonstrated higher levels of dementia knowledge and lower odds of reporting greater dementia‐related stigma compared to other ethnic groups. Previous research has highlighted disparities in timely diagnosis and access to dementia care, as well as the crucial role of knowledge and stigma in shaping help‐seeking behaviours among ethnic minority groups when facing dementia‐related concerns [26, 27, 28]. Our findings suggest that these disparities are present within minority ethnic communities even before they are personally affected by dementia. It is likely that dementia‐related stigma is perceived, anticipated, and reinforced differently across minority groups and within various sociocultural contexts [29]. This underscores the importance of integrating dementia awareness initiatives within broader public health frameworks and ensuring they are culturally competent [30, 31].

Our findings support public health campaigns, primary care and community‐driven awareness programs to enhance dementia prevention knowledge and reduce stigma among minority ethnic groups before concerns about dementia arise [30]. Improving healthcare professionals' understanding of diverse cultural conceptualizations of dementia and ageing would also be valuable in providing equitable and effective care [32, 33].

Findings align with existing evidence that dementia‐related knowledge and attitudes vary across sociocultural contexts and are shaped by exposure, education, and structural factors [14]. The association of education with knowledge of dementia risk and dementia may indicate potential avenues for intervention. Previous findings have indicated that quality of education plays a critical role in determining dementia knowledge [34]. It is estimated that Black‐White disparities in dementia would decrease by 16%–65% if differences in educational quality were eliminated [35, 36]. Future research could investigate how to increase dementia knowledge among minority ethnic groups throughout the life course, and whether early‐life educational quality contributes to ethnic disparities in later‐life brain health and dementia knowledge.

People with chronic health conditions had lower dementia knowledge and higher perceived stigma. Enhancing dementia awareness among people with chronic conditions is particularly relevant, because chronic disease management may influence dementia risk [37]. Inequalities in dementia care cannot be considered in isolation as there are structural inequalities in health that result in poorer health outcomes for people from minority ethnic groups across conditions [38]. People from minority ethnic groups experience increased rates of cardiometabolic conditions such as diabetes, hyperlipidaemia, and hypertension [39, 40]. These conditions contribute to dementia risk through vascular, metabolic, inflammatory, and physiological stress mechanisms, potentially affecting the brain both directly and through systemic organ dysfunction [41]. Therefore, incorporating dementia education within a broader health framework, particularly within chronic disease management strategies, may be beneficial for equitable prevention efforts. The NHS England secondary prevention programme covers diabetes, respiratory and cardiovascular health. Adding a focus on preventing or delaying onset of dementia to this programme could be beneficial. Many of the interventions are the same and adding the potential to reduce risk of dementia to these preventive measures would only strengthen uptake. Ensuring dementia prevention is included within all future prevention policies will ensure opportunities are not missed [19].

We did not examine the specific reasons for dementia‐related stigma. However, previous research suggests that stigma within ethnic minority populations is multifaceted [22] and may require a comprehensive approach that integrates public health initiatives, awareness‐raising among healthcare professionals and social workers, community‐led programs, and individual empowerment strategies. Considering the cultural competence of any future programmes must also consider intersectionality. We found gender‐related differences in attitudes towards dementia, with women reporting higher perceived stigma. However, only binary categories for sex were given as options which limits the generalisability of findings. Previous research has found that ethnic background may be associated with gender‐related attitudes [42, 43], although further research is needed to confirm it in the context of dementia. Further research is required to investigate stigma process and variants within the context of dementia and to explore the underlying mechanisms contributing to these differences in minority ethnic groups, including the potential influence of culturally embedded gender role or ageing perceptions [42]. Stigma may be internalized, influencing self‐efficacy and self‐worth, externally reinforced through societal norms, or shaped by a combination of both factors [22]. Understanding how dementia is perceived and how stigma is experienced is essential for developing effective interventions. In the effort to reduce stigma and promote health promotion behaviours (e.g., seeking information or help for dementia concerns) it is important to focus on both reducing stigma and increasing motivation. Although no causal relationship can be inferred from the present findings, modifying attitudes alone is unlikely to be sufficient to facilitate meaningful behavioural change [44, 45].

We found that individuals from ethnic minority groups had less personal experience and exposure to people with dementia compared to the White group. This may be attributed to the underreporting or underdiagnosis of dementia cases, lower knowledge, and stigma within these populations [27]. In immigrant families, grandparents were more likely to be living at a distance [46]. Our findings also suggest that when individuals from ethnic minority groups are exposed to someone with dementia, their knowledge of dementia risk factors may increase. This aligns with previous research indicating that contact with individuals with dementia can contribute to improved dementia knowledge [47, 48]. Future research could incorporate ethical frameworks to explore the co‐creation of culturally sensitive anti‐stigma interventions, engaging individuals with dementia and their families in public awareness initiatives. Investigating which elements of these programs are most effective and feasible for implementation could enhance efforts to reduce stigma and promote dementia awareness in diverse communities [47, 48].

We found that ethnicity, education level, profession, and chronic health conditions are associated with knowledge and attitude towards dementia. These sociodemographic factors are also associated with digital exclusion, which may be associated with differences in access to online health assessments and support services [49]. Universal digital health checks or online platforms for screening, preventive care, and support [50] may not ensure equitable access to dementia care and prevention alone. Provision for in‐person assessments, as planned for the implementation of the NHS digital health check would be key [51]. Digital preventive care has the potential to alleviate the healthcare burden by offering broad access at a relatively low cost. However, if not sustainably implemented at scale, there is a risk of the digital divide exacerbating existing healthcare inequalities [52]. To ensure equitable access to digital health tools, integrating user‐friendly designs, strengthening infrastructure such as WiFi accessibility, and implementing targeted educational initiatives may be beneficial [49]. Personalization strategies, culturally sensitive content, and incorporating human interaction could enhance trust, adherence, and engagement with digital interventions while supporting individuals in navigating digital health resources more confidently. The implementation readiness of online tools for dementia requires careful consideration to ensure their adoption by the target population and integration into real‐world healthcare settings [53].

### 5.1 Limitations

This study has important limitations. Its cross‐sectional design prevents causal inferences, and the reliance on self‐reported data may introduce biases. While the study distinguishes between major ethnic groups, it does not account for intra‐ethnic variations, including ancestral, sociocultural and linguistic differences within South Asian, Black, or Other communities, which may influence dementia‐related attitudes. Ethnic groups were combined to make the analyses tractable. Findings should therefore not be interpreted as reflecting uniform experiences within these populations. Future work should explore within‐group variation and employ more context‐sensitive classifications to better capture the diversity of experiences and determinants of health within these populations. The surveys were administered in English, potentially further limiting the inclusion of people from minority groups. Sampling bias may have led to the underrepresentation of individuals with lower educational attainment, health knowledge or limited digital access, particularly among people from minority ethnic groups. Participants in this study were predominantly from higher socioeconomic backgrounds and had higher levels of education compared to the general population of ethnic minority groups in the UK [54]. This may limit the generalizability of the findings to individuals from ethnic minority groups with lower education levels or socioeconomic status. The observed association between higher education and stigma should be interpreted with caution. The finding may reflect residual confounding or underlying differences in sample composition as people from minority ethnic groups in this sample were more educated and also experience higher stigma, although the coefficient for the association between education and stigma was small. Further research is required to better understand this association.

Outcome measures were based on a limited number of survey items and should be interpreted as proxy indicators rather than comprehensive assessments of knowledge or stigma. In particular, stigma was captured using a single disclosure‐related item. Although this item was analysed using ordinal logistic regression to better reflect its ordinal response structure, it should still be interpreted as a proxy indicator of disclosure‐related stigma rather than as a measure of the comprehensive, multidimensional nature of dementia stigma.

Due to differences in how questions were framed, we could not assess knowledge about dementia risk factors across the whole sample. Knowledge scores about dementia risk factors were lower than average knowledge scores relating to brain health, indicating the public does not necessarily equate these concepts and that messaging around dementia perhaps needs to name dementia specifically. Complete case analysis was used, which may introduce bias if missingness is not random. Although the proportion of missing data was low, the possibility of residual bias cannot be excluded. Given these limitations, future research could benefit from qualitative work to better understand the underlying differences identified, by exploring influences both related to ethnicity and other meaningful factors such as language, culture, or lived experience within and across groups.

## 6 Conclusion

This study reveals significant ethnic disparities in dementia knowledge, odds of reporting greater disclosure‐related stigma, and risk awareness. White participants demonstrated higher dementia knowledge and lower stigma, compared to other ethnic groups. Gender, education, social grade, chronic conditions, and knowing someone with dementia were contributing factors to dementia knowledge and attitudes towards dementia. To promote equity, culturally tailored education, stigma‐reduction initiatives, and accessible services should be considered as part of dementia prevention strategies. These should consider how to convey evidence‐based recommendations without stigmatising and problematising those at risk of these conditions, especially in more deprived areas where the strongest levers of change for risk reduction often lie at a societal level [55]. To increase dementia knowledge among people living with other chronic conditions, policymakers might consider how prevention could be promoted in all health and social care encounters. Primary care, social prescribers and care managers [56] might be well positioned to have conversations about promoting future health and wellbeing.

## Author Contributions

All co‐authors contributed to the conceptualisation of the study. G.A. conducted the formal analysis and drafted the manuscript. N.M. provided supervision. C.M. contributed additional resources and, together with C.C. and S.B., provided critical feedback. All co‐authors reviewed and approved the final manuscript.

## Funding

This research is funded through the NIHR Policy Research Unit in Dementia and Neurodegeneration — Queen Mary University of London, reference NIHR206110. The views expressed are those of the author(s) and not necessarily those of the NIHR or the Department of Health and Social Care. G.A. was supported by Alzheimer Nederland under grant agreement WE.15–2021‐02.

## Conflicts of Interest

The authors declare no conflicts of interest.

## Data Availability

The data supporting the findings of this study were provided by Alzheimer's Research UK and are available online at https://dementiastatistics.org/attitudes/.

## Appendix 1

**TABLE A1:** An overview of survey questions.

Variable	Rating	Survey question
Exposure	Ethnicity White (e.g., English, Welsh, Scottish, Northern Irish, British, any other White background) Asian (e.g., Pakistani, Bangladeshi, etc) Black (e.g., African, Caribbean, etc) Other= (e.g., Arab, mix, other)	Do you think of yourself as a member of any particular ethnic group? If you feel uncomfortable answering this question, please feel free to select ‘prefer not to say’’.
Confounder 1	Age, in yrs, continuous	Please state your age.
Confounder 2	Sex – binary (male = 1, female = 2)	Thinking about your gender, how do you identify?
Confounder 3	Social grade (lower = 0, higher = 1)	C2, D, E = 0, A, B, C1 = 1
Confounder 4	Education (non graduate = 0, Other (e.g., professional qualification) = 1, Graduate = 2	Please select the highest level of academic or professional qualification you have completed. If you weren't educated in the UK, please try to say the closest equivalent
Confounder 5	Having chronic condition (no = 0, yes = 1)	Do you have any of the following long‐term health conditions that impacts or limits your day‐to‐day life? (e.g., chronic pain diabetes)
Confounder 6	Knowing someone with dementia (no = 0, yes = 1)	Have you or someone you know been diagnosed as having a form of dementia such as Alzheimer's disease?
Knowledge about dementia	Ranging from 0 to 4. Higher score indicates higher knowledge.	To what extent do you agree or disagree with the following statements? Brain problems, such as dementia, are natural consequences of old age Agree = 0 Neither agree nor disagree = 1 Disagree = 2 To what extent do you agree or disagree with the following statements? There is not much you can do about protecting brain health Agree = 0 Neither agree nor disagree = 1 Disagree = 2
Stigma	Agree = 0 (low stigma), neither agree nor disagree = 1 (moderate) Disagree = 2 (high)	Please tell me the extent to which you agree or disagree? If I was diagnosed with dementia, I would feel comfortable telling people outside my own family
Knowledge about dementia risk factors	Negative = 0, Positive = 2 (ranging from 0 to 26)	Total of 13 items => HEA9—what, if anything, do you think could increase a person's risk of developing dementia?: Poor diet HEA9—what, if anything, do you think could increase a person's risk of developing dementia?: Lack of exercise HEA9—what, if anything, do you think could increase a person's risk of developing dementia?: Poor sleep HEA9—what, if anything, do you think could increase a person's risk of developing dementia?: Mental health problems HEA9—what, if anything, do you think could increase a person's risk of developing dementia?: Being less mentally active HEA9—what, if anything, do you think could increase a person's risk of developing dementia?: Loneliness/isolation/living alone/lack of social interaction HEA9—what, if anything, do you think could increase a person's risk of developing dementia?: Heavy drinking HEA9—what, if anything, do you think could increase a person's risk of developing dementia?: Smoking HEA9—what, if anything, do you think could increase a person's risk of developing dementia?: Hearing loss HEA9—what, if anything, do you think could increase a person's risk of developing dementia?: High cholesterol HEA9—what, if anything, do you think could increase a person's risk of developing dementia?: High blood pressure HEA9—what, if anything, do you think could increase a person's risk of developing dementia?: Diabetes HEA4—which, if any, of the following health conditions do you think it's possible for people to reduce their risk of developing?: Dementia
Knowledge about brain health	Negative = 0 Positive = 2 (ranging from 0 to 26)	How much of an impact, if any, do you think the following have on brain health? What you eat How much of an impact, if any, do you think the following have on brain health? Physical activity How much of an impact, if any, do you think the following have on brain health? Sleep How much of an impact, if any, do you think the following have on brain health? Mental wellbeing How much of an impact, if any, do you think the following have on brain health? Activities that challenge the brain (such as reading, playing games) How much of an impact, if any, do you think the following have on brain health? Social contact How much of an impact, if any, do you think the following have on brain health? Alcohol How much of an impact, if any, do you think the following have on brain health? Smoking How much of an impact, if any, do you think the following have on brain health? Hearing loss How much of an impact, if any, do you think the following have on brain health? Cholesterol levels How much of an impact, if any, do you think the following have on brain health? Blood pressure How much of an impact, if any, do you think the following have on brain health? Blood sugar levels Do you think people can reduce their risk of developing the following health conditions? Dementia

## Appendix 2

**TABLE A2:** Description of the socio‐demographic characteristics of the study sample in survey 1 by ethnicity.

Confounder	Ethnicity				
	White	South Asian	Black	Other	Total
	*N* = 1897	*N* = 210	*N* = 211	*N* = 183	*N* = 2530
Age					
Mean (SD)	51.78 (17.05)	37.06 (13.81)	36.32 (13.82)	39.70 (15.76)	48.34 (17.54)
Missing	49	7	8	4	79
%	1.94	0.28	0.32	0.16	3.12
Sex male	952	122	95	92	1274
%	74.73	9.58	7.46	7.22	100.00
Female	922	84	112	86	1217
%	75.76	6.90	9.20	7.07	100.00
Missing	23	4	4	5	39
%	58.97	10.26	10.26	12.82	100.00
Lower social grade	567	60	66	37	735
%	77.14	8.16	8.98	5.03	100.00
Higher social grade	1262	138	131	129	1674
%	75.39	8.24	7.83	7.71	100.00
Missing	68	12	14	17	121
%	56.20	9.92	11.57	14.05	100.00
Non education	857	71	87	68	1094
%	78.34	6.49	7.95	6.22	100.00
Other education	152	15	12	11	191
%	79.58	7.85	6.28	5.76	100.00
Graduate	854	123	111	100	1200
%	71.17	10.25	9.25	8.33	100.00
Missing	34	1	1	4	45
%	75.56	2.22	2.22	8.89	100.00
No chronic condition	1121	171	164	119	1596
%	70.24	10.71	10.28	7.46	100.00
Having chronic condition	759	37	43	56	899
%	84.43	4.12	4.78	6.23	100.00
Missing	17	2	4	8	35
%	48.57	5.71	11.43	22.86	100.00
Not knowing someone with dementia	815	142	149	104	1225
%	66.53	11.59	12.16	8.49	100.00
Knowing someone with dementia	1075	66	57	79	1290
%	83.33	5.12	4.42	6.12	100.00
Missing	7	2	5	0	15
%	46.67	13.33	33.33	0.00	100.00

**TABLE A3:** Description of the socio‐demographic characteristics of the study sample in survey 2 by ethnicity.

Confounder	South Asian	Black	Chinese	Total
	*N* = 470	*N* = 454	4 = 76	*N* = 1000
Age				
Mean (SD)	48.69 (5.52)	48.86 (5.78)	49.21 (5.93)	48.81 (5.66)
Missing	7	13	2	22
%	0.70	1.30	0.20	2.20
Sex, male	206	214	41	461
%	44.69	46.42	8.89	100.00
Female	262	239	35	536
%	48.88	44.59	6.53	100.00
Missing	2	1	0	3
%	66.67	33.33	0.00	100.00
Lower social grade	140	142	13	295
%	47.46	48.14	4.41	100
Higher social grade	330	312	63	705
%	46.81	44.26	8.94	100
Missing				
%	0	0	0	0
Non education	168	146	16	330
%	50.91	44.24	4.85	100.00
Other education	3	5	0	8
%	37.50	62.50	0.00	100.00
Graduate	299	303	60	662
%	45.17	45.77	9.06	100.00
Missing				
%	0	0	0	0
Chronic condition				
No	258	269	53	580
%	44.48	46.38	9.14	100.00
Yes	206	182	23	411
%	50.12	44.28	5.60	100.00
Missing	6	3	0	9
%	66.67	33.33	0.00	100.00
Not knowing someone with dementia	244	265	37	546
%	44.69	48.53	6.78	100.00
knowing someone with dementia	196	166	35	397
%	49.37	41.81	8.82	100.00
Missing	30	23	4	57
%	52.63	40.35	7.02	100.00

## Appendix 3

Unadjusted regression result.

Knowledge about Dementia (combined data).

Linear regression.

**Table:**

Knowledge about dementia	Coef.	St.Err.	t‐value	*p*‐value	[95% Conf	Interval]	Sig
White	0	.	.	.	.	.	
South Asian	−0.419	0.05	−8.37	0	−0.517	−0.321	***
Black	−0.112	0.051	−2.22	0.027	−0.211	−0.013	**
Other	−0.227	0.076	−3.00	0.003	−0.375	−0.079	***
Constant	3.19	0.026	122.89	0	3.139	3.241	***

Mean dependent var	3.069	SD dependent var	1.122
R‐squared	0.021	Number of obs	3402
*F*‐test	24.132	Prob > F	0.000
Akaike crit. (AIC)	10,373.097	Bayesian crit. (BIC)	10,397.625

**p* < 0.1. ***p* < 0.05. ****p* < 0.01.

Stigma (combined data).

Ordinal logistic regression.

**Table:**

Stigma	Odds ratio	Std. err.	z	*p*‐value	Lower 95% CI	Upper 95% CI
White	0	.	.	.	.	.
South Asian	1.648	0.150	5.500	< 0.001	1.379	1.969
Black	2.561	0.233	10.330	< 0.001	2.143	3.062
Other	1.613	0.213	3.630	< 0.001	1.246	2.089
/cut1	0.648	0.048	—	—	0.554	0.741
/cut2	1.023	0.050	—	—	0.926	1.121

Odds ratios above 1 indicate higher odds of reporting greater stigma. White participants were the reference group.

**Table:**

Stigma	Coef.	St.Err.	t‐value	*p*‐value	[95% Conf	Interval]	Sig
White	0	.	.	.	.	.	
South Asian	0.249	0.041	6.08	0	0.169	0.33	***
Black	0.456	0.041	11.04	0	0.375	0.537	***
Other	0.234	0.06	3.87	0	0.115	0.353	***
Constant	0.593	0.021	28.17	0	0.552	0.635	***

Mean dependent var	0.746	SD dependent var	0.922
R‐squared	0.038	Number of obs	3412
*F*‐test	44.993	Prob > F	0.000
Akaike crit. (AIC)	9006.723	Bayesian crit. (BIC)	9031.263

**p* < 0.1. ***p* < 0.05. ****p* < 0.01.

Knowledge about dementia risk factors (Survey 1, mix group).

Linear regression.

**Table:**

Risk knowledge	Coef.	St.Err.	t‐value	*p*‐value	[95% Conf	Interval]	Sig
White	0	.	.	.	.	.	
South Asian	12.827	0.316	40.61	0	12.208	13.446	***
Black	12.393	0.319	38.91	0	11.768	13.017	***
Other	5.058	0.468	10.80	0	4.14	5.976	***
Constant	2.455	0.162	15.13	0	2.137	2.774	***

Mean dependent var	7.675	SD dependent var	9.257
R‐squared	0.418	Number of obs	3501
*F*‐test	835.898	Prob > F	0.000
Akaike crit. (AIC)	23,631.845	Bayesian crit. (BIC)	23,656.488

**p* < 0.1. ***p* < 0.05. ****p* < 0.01.
